# Assessment of Insulin Injection Practice among Diabetes Patients in a Tertiary Healthcare Centre in Nepal: A Preliminary Study

**DOI:** 10.1155/2017/8648316

**Published:** 2017-12-03

**Authors:** Ramesh Sharma Poudel, Shakti Shrestha, Rano Mal Piryani, Bijaya Basyal, Kalpana Kaucha, Shital Adhikari

**Affiliations:** ^1^Hospital Pharmacy, Chitwan Medical College Teaching Hospital, Chitwan, Nepal; ^2^Department of Pharmacy, Shree Medical and Technical College, Chitwan, Nepal; ^3^Department of Internal Medicine, Chitwan Medical College Teaching Hospital, Chitwan, Nepal

## Abstract

**Introduction:**

Proper insulin injection practice is essential for better diabetic control. This study aims to assess the insulin injection practice of patients with diabetes.

**Materials and Methods:**

A cross-sectional study was conducted at Chitwan Medical College Teaching Hospital, Bharatpur, Nepal, from February 2017 to May 2017. Patients injecting insulin through insulin pens (*n* = 43) for a minimum of 4 weeks were consecutively recruited. Patients' baseline characteristics, current insulin injection technique, insulin transportation practice, complications of insulin injection, disposal practice of used needle, and acceptability of insulin were recorded. Descriptive statistics were performed using IBM-SPSS 20.0.

**Results:**

The insulin injection technique of patients and their relatives was inadequate. The majority of patients and their relatives (25, 58.1%) mentioned that they transport their insulin cartridge without maintaining cold chain. Thirteen patients (30.2%, *n* = 43) reported complications of insulin injection and the most common complication among those patients was bruising (10, 76.9%, *n* = 13). Almost all patients disposed the used needle improperly, and the common method was disposing the needle in a dustbin and then transferring to municipal waste disposal vehicle. Insulin was accepted by just 16 (37.2%) patients.

**Conclusion:**

There was a significant gap between the insulin delivery recommendation through insulin pen and current insulin injection practice.

## 1. Introduction

Diabetes mellitus (DM) affects 415 million people worldwide with an estimated prevalence of 9.1% in 2015 and is predicted to increase to 10% by 2040 [1]. Low- and middle-income countries bear a burden of about three quarters of diabetic population [1]. The prevalence of diabetes in Nepal is increasing [2, 3] and is projected to reach 17.49% by 2020 [3]. Insulin is the indispensable component of the management of DM [4], and the proportions of patients using insulin vary from country to country [5, 6]. Unfortunately, reliable statistics on proportion of diabetes patients using insulin are lacking from Nepal. Correct insulin injection technique is essential for better diabetic control [7]. However, one of the large multinational surveys (*n* = 13,289) in 42 countries showed that the patients' insulin injection technique was inappropriate [8]. Studies in our neighboring countries, India and China, also showed a significant gap between the insulin administration guidelines and insulin injection technique [9–11]. Incorrect insulin injection techniques are not only associated with higher consumption of insulin and higher glycated hemoglobin values but also are responsible for higher frequencies of unexpected hypoglycemia and glucose variability [8]. Moreover, it has been also reported that faulty injection technique can cause insulin allergy [12, 13]. Furthermore, there are evidences suggesting suboptimal disposal of needle after use [8, 9], thereby increasing the risk of transmitting blood-borne infections [14]. There is paucity of data regarding insulin injection practice from Nepal where insulin is perceived as the “last option” available for treatment [15]. Hospital pharmacy services in Nepal are at early stage, and therefore, pharmaceutical care might be suboptimal [16, 17]. Additionally, education mostly provided by Nepalese community pharmacies are also inadequate [18, 19]. Considering this scenario, insulin injection technique might be poor/inadequate among Nepalese patients with DM. Therefore, it seemed rational to assess insulin injection practice of patients with diabetes, the objective of our study.

## 2. Materials and Methods

### 2.1. Study Design

This cross-sectional study was conducted at the Medication Counseling Centre of Chitwan Medical College Teaching Hospital (CMCTH), Bharatpur, Nepal, from February 2017 to May 2017. Ethical approval of this study was obtained from the Institutional Review Committee of the Chitwan Medical College Teaching Hospital. This study was designed to assess the insulin injection practice among patients with DM. Insulin injection practice was assessed by a registered pharmacist. The pharmacist was educated and trained in insulin injection practice according to the reference of Forum for Injection Technique and Therapy: Expert Recommendations [20] by the Health Professionals Education and Research Centre of CMCTH. In addition, the pharmacist was urged to use drug information leaflets enclosed inside the insulin and insulin pen device.

### 2.2. Study Population

All patients with DM who have been injecting insulin through insulin pen for a minimum of 4 weeks were consecutively recruited. Those using syringes for injecting insulin and unwilling to participate in this study were excluded. A total of 43 patients were included during study period.

### 2.3. Assessment of Insulin Injection Technique

The pharmacist made appointments with the patients and assessed their eligibility for the study. Patients who met the inclusion criteria of the study were requested to participate in the study. They were well informed about the study, and a written informed consent was obtained from either patients or their relatives. The insulin injection practice was assessed according to the reference of Forum for Injection Technique and Therapy: Expert Recommendations [20]. The questionnaires included two sections. Section one consisted of baseline characteristics: age, sex, regional area, residential area, educational status, employment status, diabetes type, duration of diabetes, duration of insulin therapy, number of insulin use, administration (self or by others), currently used insulin, perceived confidence in insulin pen, and initial insulin pen educator. Section two included (a) current insulin injection technique: storage of insulin, time gap between injection and meal, hand washing prior to injection, cleaning of injection site, mixing of insulin prior to use, method of mixing, priming of pen before injection, injection site, the use of skin fold, insulin-injecting angle, needle holding time under the skin, massage of injection site, needle length, average frequency of one needle use, and site rotation, (b) insulin transportation practice; (c) complications of insulin injection; (d) disposal practice of used needle; and (e) acceptability of insulin. A structured interview was conducted to obtain the baseline characteristics and insulin injection practice; however, local site complications were examined by the pharmacist. Two parameters/questions (mixing of insulin before injection and mixing process) were skipped whenever the patient was using basal insulin and lispro insulin. The mixing process was categorized as correct or incorrect based on the patient reports. In case of patients using two different types of insulin, the technique of premix insulin was assessed. Those who had incorrect insulin injection practice were educated and counseled about correct insulin injection by a pharmacist. The patients and their relatives were also allowed to ask questions for further clarification. However, the effectiveness of pharmacist counseling was not evaluated in this study.

### 2.4. Statistical Analysis

Descriptive statistics were performed using IBM-SPSS 20.0 (IBM Corporation, Armonk, NY, USA). Shapiro-Wilk test was used to determine the normality of numeric variables. Median and interquartile range (IQR) were calculated for all the numeric variables which failed to follow normal distribution. The categorical variables were presented as the frequency and their respective percentage.

## 3. Results

Forty-three patients completed the study. The median (IQR) age of the participants was 55 (49–63) years. The majority (55.8%) of them were females and about 56% were from suburban areas. Most of the participants were illiterate (58.1%) and unemployed (67.4%). Forty-one (95.3%) patients were suffering from type II DM and two (4.7%) from type I DM. The median (IQR) duration of DM was 7 (3–10) years while median (IQR) duration of insulin therapy was 0.91 (0.80–3) years. More than four-fifths of the participants used a single type of insulin for the management of DM. The premixed insulin (60.5%) was the most common type of insulin used in this study. Just over two-fifths of the patients were dependent upon others to inject their insulin while nearly two-thirds were not sure about their insulin injection technique. The majority of the participants (53.5%) reported that they received instruction from clinicians and 37.2% received advice from a nurse (Table 1).

The assessment of insulin injection technique and insulin pen storage practice revealed that twenty (46.5%) patients were storing their insulin pen (insulin cartridge inside) at room temperature and an equal number of patients kept their insulin pen inside refrigerator while three (7%) patients stored their insulin pen in a clay pot. The median (IQR) time gap between injection and meal was 15 (15–30) minutes. Thirty-one (72.1%) patients or their relatives followed the practice of hand washing before injection. Cleaning of the injection site before insulin administration was not practiced by 30 (69.8%) participants. Of 30 patients using premix insulin, just 12 (40% of 30) patients were mixing the insulin before administration but only five (41.7% of 12) patients mixed the insulin properly. None of the patients or relatives primed insulin pen before injection. The thigh was the most common site of injection of insulin (19, 44.2%) followed by abdomen (16, 37.2%) and using both sites (thigh and abdomen) (8, 18.6%). Nearly three quarters (32, 74.42) of the patients made a skin fold while injecting the insulin and 34 (79.1%) patients injected insulin at nearly 90° angle. Nearly half of the patients or their relatives (20, 46.5%) stated that they wait less than 5 seconds after completely inserting the thumb bottom before withdrawaling insulin needle from the skin. About one in five patients (9, 20.9%) mentioned that they massage the injection site after administration of insulin. The proportion of patients using 5 mm, 6 mm, and 8 mm needles was 11 (25.6%), 28 (65.1%), and four (9.3%), respectively. All the patients were known to use a single needle more than once for injecting insulin and the median (IQR) number of single needle use was 16 (12–30) times. Thirty (69.8%) participants were known to rotate the injection sites while injecting insulin.

The majority of patients (25, 58.1%) and their relative mentioned that they transport insulin without maintaining the cold chain. Nearly one-third of (13, 30.2%) them reported complication of insulin injection technique and most common complication was bruising (10, 76.9%). Other complications were bleeding (1, 7.69%), pain (1, 7.69%), and scaring (1, 7.69%). Insulin was well accepted by 16 (37.2%) patients.

Just about half of the participants reported that they disposed their needles in the dustbin and then transferred them to the municipal waste disposal vehicle. Other common methods of disposal included throwing used needles in an isolated place and burning them. Interestingly, one patient collected the used needles in a separate container for 15 years and did not know about methods of disposal. Similarly, another patient collected used needles in a plastic container and kept in the refrigerator for 3 months (Table 2).

## 4. Discussion

Correct insulin injection technique is crucial for better glycemic control [7]. However, our study showed significant gaps between insulin delivery recommendations and current insulin injection practice in Nepalese patients with diabetes. The majority of patients were storing their insulin pens (insulin cartridge inside) either at room temperature or in the refrigerator. The insulin pen in use (insulin cartridge inside) can be stored at room temperature (15–25°C) for 30 days [20]. However, the room temperature in many parts of Nepal, particularly in the Terai belt, exceeds 25°C especially during summer season. The storage of insulin (regular and biphasic) at 32°C and 37°C for 28 days has shown 14–18% reduction in the potency of insulin [21]. Similarly, opened (in-use) cartridge should not be refrigerated when installed in insulin pen [22]. It has been known that variation in temperature leads to accumulation of air in the pen which inversely affects insulin delivery at its predefined time [23]. The comparatively better option would be either storing in-use insulin pen in an earthen pot or in a cooling bag. The median (IQR) time gap between injection and meal was 15 (15–30) minutes irrespective of the type of insulin. The guidelines recommend maintaining an injection-meal interval of 30 min when injecting regular insulin in abdomen and 45 min in other injection sites [20]. However, there are studies which have shown no change in HbA1c level with variation in injection-meal time [24, 25]. The majority of our participants who were from noninstitutionalized setting (home) did not clean the injection site before insulin administration. It has been recommended that insulin should be administered into clean sites using clean hands [20], and cleaning of injection site is usually not required when injections are given in noninstitutional settings such as homes, restaurants, and workplaces [20]. But nearly three quarters of patients or their relatives in our study followed the practice of hand washing before injection. Three-fifths of premix insulin users did not mix their insulin prior to administration, and there was a lower number of patients who correctly mixed insulin. Not mixing or inadequate mixing of premix insulin can alter the insulin concentration and vary the clinical response [20]. Also, it has been associated with higher consumption of insulin [8]. It has been recommended that insulin pens should be primed prior to administration of insulin in order to ensure free and unobstructed flow [20]. But none of the patients or their relatives primed insulin pen before injection; however, the majority of patients or their relatives primed during changing of insulin cartridge and pen needle. Priming is essential to ensure that newly used needle is working properly and air bubbles are removed, so that insulin delivery is not altered [23, 26]. In our study, all participants injected insulin in the thigh or/and abdomen. Others studies also reported the abdomen and thigh as most common sites of injection [8–10]. The guideline suggests that patients should count slowly from 1 and reach 5 to 10, depending on the dose of insulin, after completely pushing the thumb bottom before the withdrawal of the needle from the skin [20]. A higher dose of insulin may also contribute to longer transit time of insulin [27]. Therefore, some patients may need to count past 10, especially when giving higher doses. This is necessary to prevent medication leakage and to get the full dose. Around half of the patients in our study hold the needle beneath skin for 5–10 seconds after completely inserting the thumb bottom. Our results were better than those reported in other studies [9, 10]. It is recommended that the injection site should not be massaged after injection. However, a fifth of total patients in our study massaged injection sites after the administration of insulin. Shorter insulin pen needles (4 mm) are much safer, less painful, and more efficacious, hence better tolerated [28], and there is lack of evidence for recommending needle longer than 6 mm [20]. This recommendation is compatible with our finding as the majority of patients used either 5 mm or 6 mm needles. However, some patients were known to use 8 mm needle. This is probably due to the unavailability of shorter needles in their locality. In India, recent evidence suggests that approximately a quarter of patients still use 12.7 mm needles and a high proportion of patients use 8 mm needle [9]. All the patients were found to use same needle more than once and the median (IQR) use was 16 (12–30) times in our study. But it has been recommended that needle should not be reused [20]. A national survey in India reported that 92.5% patients reused pen needle for at least 2 times to more than 10 times [10]. Another study reported that Indian patients with DM used each needle for an average of six times to inject insulin [9]. The most common reasons for the reuse of needles were to save money and for convenience [8, 11]. In Nepal, the high frequency of needle reuse might be due to financial constraint and there is no reimbursement system for needles. Making needles cheaper or distributing needles to patients free of cost can be useful means to overcome this problem. The reuse of needles can lead to loss of sterility and lubrication or damage to the needle tip by bending or breakage [6, 20, 26]. Furthermore, needle reuse also increases the risk of contamination and infection, injection pain, injection site irritation, or damage; the risk of the needle breaking off and remaining in the tissue [6, 20, 26]; and the development of lipohypertrophy [29, 30]. Lipohypertrophy can be avoided through systematic rotation of injection site [20, 29]. This practice was followed by around 70% of patients and insulin was well accepted by just over one-third of the patients in our study. One of the prime reasons for rejecting the insulin therapy is related to psychology (e.g., anxiety) of patients [20]. Moreover, Nepalese patients with diabetes have been known to perceive insulin as the “last option” available for treatment [15]. Almost all patients disposed used needles improperly in our study. The most common methods included transfer of used needles to municipal waste disposal vehicles, throwing them in isolated places, and burning. These situations are a clear indication of lack of awareness on needle disposal together with absent of regulatory requirement. Similarly, majority of the Indian patients were known to throw the needle and syringes directly into the garbage and public drainage system [9]. Other studies also reported improper disposal of used needles [8, 10].

Healthcare professionals should educate and reinforce patients and their relatives about the correct use of insulin pens during their first visit and subsequent follow-ups. They can play a significant role in safe and efficient use of insulin pens in diabetic patients [31]. Our study had few limitations. This was a single center study; therefore, it might not sufficiently generalize the insulin injection practices in Nepal. However, this is probably the first evidence from Nepal to add value to the existing literature. Also, this study was conducted based on reports from the patients or their relatives which bear a potential information bias. Direct observation of the injection technique by a trained healthcare professional would avoid such bias.

## 5. Conclusion

There was a significant gap between the insulin delivery recommendation through insulin pen and current insulin injection practice. Education and counseling on proper insulin pen injection technique should be provided to patients with diabetes using insulin in Nepal.

## Figures and Tables

**Table 1:** Baseline characteristics of the patients.

Variables	Category	*n* (%)
Age (years)^∗^		55 (49–63)
Sex	Male	19 (44.2)
	Female	24 (55.8)
Regional area	Pahada	7 (16.3)
	Tarai	36 (83.7)
Residential area	Suburban	24 (55.8)
	Rural	19 (44.2)
Educational status	Literate/educated	18 (41.9)
	Illiterate/uneducated	25 (58.1)
Employment status	Employed	14 (32.6)
	Unemployed	29 (67.4)
Diabetes type	Type I	2 (4.7)
	Type II	41 (95.3)
Duration of diabetes (years)^∗^		7 (3–10)
Duration of Insulin therapy (years)^∗^		0.91 (0.80–3)
Type of insulin used	One	38 (88.4)
	Two	5 (11.6)
Administration of insulin	Self	25 (58.1)
	By others	18 (41.9)
Currently used insulin	Premix insulin	26 (60.5)
	Basal insulin	12 (27.9)
	Premix and basal insulin	4 (9.3)
	Basal and lispro insulin	1 (2.3)
Do you feel your IIT is correct?	Yes	17 (39.5)
	Not sure	26 (60.5)
Who instruct you about IIT?	Clinicians/physician	23 (53.5)
	Nurse	16 (37.2)
	Pharmacy personnel	3 (7.0)
	Others	1 (2.3)

IIT: insulin injection technique. ^∗^Median [interquartile range (IQR)].

**Table 2:** Disposal practice of used needle.

Disposal practice	*n* (%)
Isolated place (bamboo tree/unused land/cave/jungle/hole of tree/near tree)	9 (20.9)
Collect in dustbin with other waste and transfer to municipal waste disposal vehicle	21 (48.8)
Others (collect in separate container since 15 years/collect in box and kept in refrigerator/burry/thrown in toilet and river/kept below large stone)	8 (18.6)
Collect and burn	4 (9.3)
Properly disposed	1 (2.3)
